# AGE-FRIENDLY HEALTH SYSTEM “4MS STOP-AND-WATCH” PROGRAM: A CNA EDUCATION INITIATIVE IN LONG-TERM CARE

**DOI:** 10.1093/geroni/igae098.2168

**Published:** 2024-12-31

**Authors:** Susan Elliott

**Affiliations:** Saint Louis University School of Medicine, St. Louis, Missouri, United States

## Abstract

The valuable contributions of Certified Nursing Assistants (CNAs) working in the longterm care setting are often overlooked, particularly in the implementation of Age-Friendly Health System (AFHS) practices. The “4Ms Stop-and-Watch” initiative was developed to integrate CNAs into the AFHS transformation of a post-acute longterm care facility. With support of the facility administration, our team created didactic and experiential learning opportunities in which all facility CNA staff were invited to participate. CNAs first attended a presentation on the AFHS concepts and the 4Ms framework with a description of the “Stop-and-Watch” program provided. Participants were directed to use a paper form to identify and address at least one 4M during each shift. The information was then entered into an online questionnaire before the end of their shift. The CNA identified which of the 4Ms was addressed and the action taken (e.g., inform nursing supervisor). Survey results were compiled and analyzed to determine effectiveness of education, grasp of 4Ms assessment and intervention, and trends in resident encounters. Feedback was provided to CNAs and facility administration. The presentation will provide detailed information to enable facilities to develop a “4Ms Stop-and-Watch” program to optimize the competency and contributions of the direct care staff.

